# Multiple mutations in the nicotinic acetylcholine receptor *Ccα6* gene associated with resistance to spinosad in medfly

**DOI:** 10.1038/s41598-019-38681-w

**Published:** 2019-02-27

**Authors:** Enric Ureña, Ana Guillem-Amat, Francisco Couso-Ferrer, Beatriz Beroiz, Nathalia Perera, Elena López-Errasquín, Pedro Castañera, Félix Ortego, Pedro Hernández-Crespo

**Affiliations:** 1 Departamento de Biotecnología Microbiana y de Plantas, Centro de Investigaciones Biológicas, CSIC, Madrid, Spain; 2 Present Address: Institute of Healthy Ageing, Department of Genetics, Evolution and Environment, University College London, Gower St, London, WC1E 6BT UK; 3 Universidad Politecnica de Madrid, Madrid, Spain

## Abstract

Spinosad is an insecticide widely used for the control of insect pest species, including Mediterranean fruit fly, *Ceratitis capitata*. Its target site is the α6 subunit of the nicotinic acetylcholine receptors, and different mutations in this subunit confer resistance to spinosad in diverse insect species. The insect *α6* gene contains 12 exons, with mutually exclusive versions of exons 3 (3a, 3b) and 8 (8a, 8b, 8c). We report here the selection of a medfly strain highly resistant to spinosad, JW-100 s, and we identify three recessive *Ccα6* mutant alleles in the JW-100 s population: (i) *Ccα6*^3aQ68*^ containing a point mutation that generates a premature stop codon on exon 3a (3aQ68*); (ii) *Ccα6*^*3aAG*>*AT*^ containing a point mutation in the 5′ splicing site of exon 3a (3aAG > AT); and (iii) *Ccα6*^3aQ68*-K352*^ that contains the mutation 3aQ68* and another point mutation on exon 10 (K352*). Though our analysis of the susceptibility to spinosad in field populations indicates that resistance has not yet evolved, a better understanding of the mechanism of action of spinosad is essential to implement sustainable management practices to avoid the development of resistance in field populations.

## Introduction

Spinosad is a natural product mixture of two compounds, spinosyn A and spinosyn D, produced by the bacteria *Saccharopolyspora spinosa* and used as insecticide since 1997^1^. It affects the insect central nervous system causing involuntary neuronal excitation that produces muscle contraction, tremors, paralysis and death^2^. Spinosad acts in insects as an allosteric agonist of acetylcholine by binding to nicotinic acetylcholine receptors (nAChRs) that function as neurotransmitter ligand-gated ion channels^3^. nAChRs are composed by the homomeric or heteromeric assembly of five ligand-binding nAChR subunits circularly gathered to form the channel. A typical nAChR subunit contains an extracellular N-terminal domain including six loops (A-F) involved in the acetylcholine binding site, four transmembrane segments (TM1–4, of which TM2 is part of the receptor channel pore), and a large intracellular domain (between TM3 and TM4)^4^. Insect species have between 10–16 subunits in their genome, 10 in *Drosophila melanogaster* (α1–7 and β1–3), and 11 in *C*. *capitata* (α1–8 and β1–3)^5^. The specific binding of spinosad to α6 subunits is believed to mediate death in insects^6–8^.

**Figure 1:**
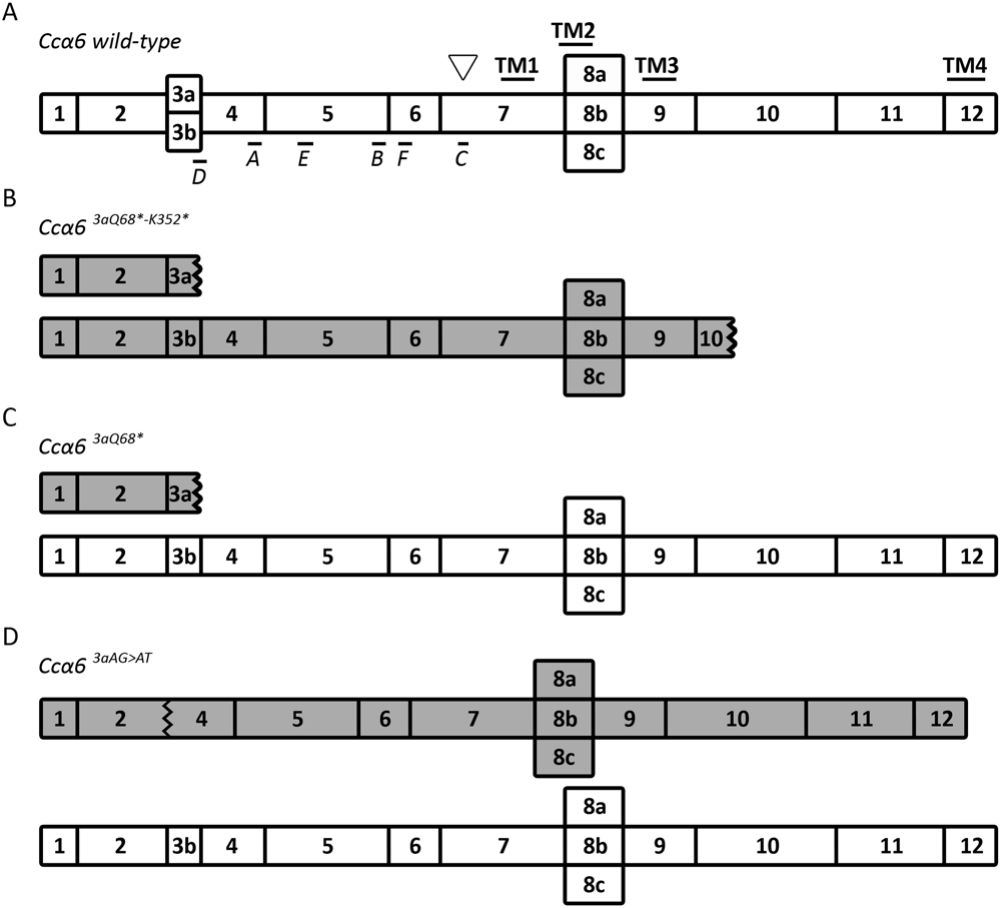
Schematic representation of the potential isoforms coded by the *Ccα6* alleles. Numbered boxes represent the exons. White figures refer to full-length wild-type isoforms, while grey figures indicate truncated isoforms. (**A**) Isoforms produced by the *Ccα6 wild-type*, with the alternative exons 3a/3b and 8a/8b/8c; TM, transmembrane domains; A-F, ligand-binding loops; inverted triangle, signature cysteines for nAChR α subunits. (**B**) Truncated isoforms produced by *Ccα6*^3aQ68*-K352*^. (**C**) Isoforms coded by the *Ccα6*^3aQ68*^. (**D**) Isoforms produced by *Ccα6*^*3aAG*>*AT*^.

nAChR α6 subunits show a high degree of conservation both in amino acid identity and genomic structure among insect species and also with the α7 subunits in vertebrates^9^. The insect *α6* gene contains 12 different exons (exon 1–12), with two variants of exon 3 (exon 3a and exon 3b) and generally three variants of exon 8 (exon 8a, 8b and 8c) (Fig. 1A). These mutually exclusive variants of exon 3 and exon 8, together with frequent A-to-I pre-mRNA editing, confers the capacity to generate a huge diversity of mRNA products. Interestingly, exon 3 contains the acetylcholine binding loop D, exon 8 includes part of the TM2 domain involved in the formation of the pore, and some of the editing sites described in *α6* are located in the proximity of the acetylcholine binding pocket^10,11^. However, whether this *α6* mRNA diversity leads to functionally distinct receptors and/or affect the interaction with spinosad remains elusive.

High levels of spinosad resistance in the field have been recorded in several pest species including the American serpentine leafminer *Liriomyza trifolii*^12^, the diamondback moth *Plutella xylostella*^13,14^, the western flower thrips *Frankliniella occidentalis*^15^, the beet armyworm *Spodoptera exigua*^16^, the Oriental leafworm moth *Spodoptera litura*^17^, the onion thrips *Thrips tabaci*^18^, the tiger mosquito *Aedes albopictus*^19^, and the tomato borer *Tuta absoluta*^20^. Moreover, selection in the laboratory has resulted in several highly resistant insect strains including the fruit fly *D*. *melanogaster* and the oriental fruit fly *Bactrocera dorsalis*^21^. Studies on *D*. *melanogaster* have found that nAChR *α6* subunit knock outs, point mutations generating premature stop codons, and amino acid changes next to the Cys-loop motif result in resistance to spinosad^6,7,22,23^. Resistance to spinosad has also been associated with generation of truncated *α6* transcripts or amino acid changes due to missplicings, insertions, deletions and point mutations in *P*. *xylostella*^24–26^, *T*. *absoluta*^20,27^, *F*. *occidentalis*^28^, the melon thrips *Thrips palmi*^29^, and *B*. *dorsalis*^30^.

At present, spinosad baits are widely used for the control of the Mediterranean fruit fly (medfly) *Ceratitis capitata* (Wiedemann) (Diptera: Tephritidae) in Spain. Other insecticides commonly used against medfly are lambda-cyhalothrin in bait sprays and deltamethrin in lure and kill traps. Medfly causes a severe economic impact in many areas dedicated to fruit production due to direct damage on fruit and to the imposition of quarantine restrictions in the exportation to medfly-free areas. Thus, intensive control measurements have been performed, including the insecticides described above and malathion before its withdrawal in the EU in 2009. Most probably as consequence of their intensive use, resistance to malathion^31,32^ and lambda-cyhalothrin^33^ has evolved in the field. In this context, there is need for implementing measurements to assure the sustainability of the use of spinosad and to counteract resistance evolved for other insecticides. The identification of resistance mechanisms and the development of specific diagnostic tools for the early detection of resistance is essential for the implementation of appropriate resistance management strategies^34^.

Here, we report data on the susceptibility to spinosad in twelve Spanish medfly populations indicating that resistance has not yet evolved in the field. In addition, we have obtained a laboratory strain highly resistant to spinosad and demonstrate that resistance in these flies is linked to recessive mutant alleles of the *α6* nAChR gene of medfly (*Ccα6*). Our data highlights the plasticity of the *Ccα6* gene to acquire non-lethal mutations, and suggests that mutations affecting the exon 3a could be sufficient to confer high levels of resistance to spinosad.

## Materials and Methods

### Field sampling

Fruit infested with *C*. *capitata* larvae were collected from commercial orchards at different localities in Spain between 2007 and 2015 (Supplementary Information, Fig. S1 and Table S1). The infested fruits were placed in ventilated plastic boxes in an environmentally controlled rearing room, at a photoperiod of 16 h light and 8 h dark, and a temperature of 26 ± 2 °C (light) and 22 ± 2 °C (dark), until pupation occurred. Emerged adults were collected daily for bioassays and fed with artificial diet (4:1:0.1, glass sucrose:hydrolyzed yeast:water) and water *ad libitum*.

### Laboratory strains

All strains were reared in the laboratory as described in Magaña *et al*. (2007)^31^. The laboratory strain C was established from *C*. *capitata* collected at non-treated experimental fields of the Instituto Valenciano de Investigaciones Agrarias (València, Spain) in 2001. It is maintained in our laboratory without exposure to insecticides. The malathion-resistant strain W-4 Km and the lambda-cyhalothrin resistant strain W-1Kλ are spinosad-susceptible strains maintained under regular selection in the laboratory, as previously reported^33,35^. The spinosad resistant strain JW-100 s derives from individuals collected in Xàbia in 2007 (Supplementary Information, Table S1), and has been obtained by laboratory selection performed by treating the adult rearing diet with spinosad: 1 to 10 ppm for 48 h from generations 1 to 29; 100 ppm for 48 h from generation 30 to 49; 100 ppm for 72 h from generation 50. From generation 24, adults were starved for 24 h before selection. At the beginning of the selection process field derived adults laid eggs on apples that were introduced in the rearing cages. At generation 10, reciprocal crosses were performed between the spinosad selected population and the malathion resistant strain W-4 Km with the objective of adapting the spinosad resistant strain to lay eggs on the net of the rearing cages, facilitating the rearing and selection processes.

### Chemicals

Insecticides used were spinosad (Spintor Cebo, 0.024% p/v CB; and 88% technical grade, Dow AgroSciences LLC, Indianapolis, IN), imidacloprid (Confidor 20LS, 20% p/v SL, Bayer CropScience, S.L., València, Spain), fipronil (97.5% technical, Sigma-Aldrich, St. Louis, USA), malathion (Agromalathion, 50% p/v EC, Agrofit S. Coop., València, Spain) and lambda-cyhalothrin (Karate Zeon, 10% p/v CS, SyngentaAgro S.A., Madrid, Spain). The synergists assayed were piperonyl butoxide (PBO; 90% technical, Aldrich, Milwaukee, WI), S,S,S-tributyl phosphorotrithioate (DEF; 98% technical, Chem Service, West Chester, PA), and diethyl maleate (DEM, 97% technical, Aldrich).

### Bioassays

Feeding bioassays were performed by confining ten to fifteen adults (3–5 days old) in ventilated plastic dishes (89 mm in diameter, 23 mm in height) containing water and rearing diet with the appropriate concentration of the insecticide. Adults from C and JW-100 s strains were starved for 24 h before treatments, whereas adults from field populations were tested without a previous starving. In all cases, 3–4 replicates were performed for each concentration. Untreated controls consisted of rearing diet mixed with water. Depending on sample size, concentration-mortality responses or discriminating concentrations were tested for field populations. Four to seven different concentrations which resulted in >0% and <100% mortality were used to obtain the concentration-mortality curves. Adult flies were kept in an environmentally controlled chamber (Sanyo MLR-350-H, Sanyo, Japan), at 25 ± 1 °C and 16 h light and 8 h dark photoperiod. Mortality was recorded after 48 h. Flies were considered dead if they were ataxic.

For topical application, adult flies (3–5 days old) were anesthetized with CO_2_ and treated with a 0.5 μl drop of insecticide solution in acetone or only acetone (used as control) on the dorsal thorax by using an automatic microapplicator 900× (Burkard Manufacturing Co., Hertfordshire, United Kingdom). Three to four replicates of 10–15 adults per dose (calculated as μg of insecticide per g of fresh weight of insect, assuming an average weight of 10 mg) were performed. After topical treatment, insects were placed in the ventilated plastic dish containing water and rearing diet. The mortality was recorded after 48 h.

The synergists PBO, DEF and DEM were diluted in acetone and applied topically to adult flies as described above. The doses applied (0.5 μg PBO, 1 μg DEF or 1 μg of DEM per insect) showed no mortality on adults. Acetone was used as a control. After 2 h, the flies were treated topically with spinosad as previously described. The mortality was recorded after 48 h. For all bioassays performed, alive and dead individuals were frozen with liquid nitrogen and stored at −80 °C.

### Reciprocal crosses

Adults of the C and JW-100 s strains were collected and their sex determined immediately after adult emergence. Males and females from each strain were placed separately into ventilated plastic dishes and maintained with water and rearing diet for two days. Reciprocal crosses between virgin individuals (100 C males x 100 JW-100 s females; and 100 JW-100 s males x 100 C females) were performed to obtain the F1 generation. The progeny of both reciprocal crosses was kept in the absence of selection pressure at standard conditions to produce the F2 generation. The susceptibility to spinosad in adults of the F1 and F2 generations was tested by feeding bioassays using concentration-mortality response for F1 and a discriminating concentration of 10 ppm for F2.

### Data analysis

Mortality data were used to estimate the concentrations/doses needed to cause 50% mortality (LC_50_ or LD_50_) by probit analysis using the computer program POLO-PC (LeOra Software14), which automatically corrects for control mortality using Abbott’s transformation^36^. Resistance ratios (*RR* = *LC*_50_ *(field or lab strain)/LC*_50_ *(C strain)*) and synergistic ratios (*SR* = *LD*_50_ *(synergist* −*)/LD*_50_ *(synergist*+)) were considered significant if their 95% fiducial limits did not include 1^37^. Mortality data when using discriminating concentrations were subjected to arcsine square root transformation and compared by ANOVA followed by Dunnett´s test. Abbott’s formula was used to correct mortality data for natural (non-treated) response^36^. The dominance value (D_LC_) of JW-100 s resistance was calculated using the formula *D*_*LC*_ = *{[(2 logLC*_50_*F1* −*logLC*_50_*P1 −logLC*_50_*P2)/(logLC*_50_*P1* −*logLC*_50_*P2)]*+*1}/2*; where P1 and P2 corresponded to parental strains JW-100 s and C, respectively^38^. Values range between 0 for completely recessive and 1 for completely dominant.

### RNA extraction, RT-PCR and sequencing of *Ccα6* from JW-100 s individuals

Total RNA was extracted from heads of JW-100 s adults using TRIzol^®^ Reagent following manufacturer’s instructions (Life Technologies, Carlsbad, CA, USA). RNA precipitation was improved by adding 20 μg/μl UltraPure glycogen (Life Technologies) and dissolved in RNAsecure Resuspension Solution (Life Technologies) followed by incubation at 60 °C for 10 min. RNA quantification was performed using Nanodrop ND-1000 spectrophotometer (Thermo Fisher Scientific, Waltham, MA, USA). Prior to reverse transcription, 2 μg of RNA was incubated with RQ1 DNase (Promega, Madison, WI, USA) at 37 °C for 35 min to eliminate genomic DNA, and the reaction was stopped by incubation with 1 μl of RQ1 DNase Stop solution (Promega) at 70 °C for 10 min. Reverse transcription was carried out using the RevertAid H Minus First Strand cDNA Synthesis Kit (Thermo Fisher Scientific). The cDNA obtained was diluted with nuclease-free water and stored at −20 °C.

*Ccα6* codifying region was amplified through PCR in a volume of 25 μl using 0.4 μM of forward and reverse oligonucleotides (FnACh6-ex1, 5′-TCGCTGTTTGCCGTGTTGATCTTT-3′; and RnACh6-ex12, 5′-TTGGACTATTATGTGCGGAGCTGA-3′) (Sigma-Aldrich), 1.4 U of Expand High Fidelity DNA polymerase (Expand High Fidelity PCR System, Roche), 10x PCR Buffer II, 2.5 mM MgCl_2_, 0.8 mM dNTPs (Thermo Scientific), and 4 μl of the corresponding cDNA as template. PCR conditions were as follows: an initial denaturation step at 95 °C for 5 min; 40 cycles of 95 °C for 30 s, 60 °C for 30 s, and 72 °C for 2 min (with an increase of 1 s for every cycle); and a final step of 72 °C for 7 min for fully extension. PCR products were analysed by electrophoresis on 1% agarose gel (Agarose D2, Conda Pronadisa) and the bands of interest were purified from the gel with Ultrafree^®^-DA Centrifugal Filter Units (Millipore, Ireland) following manufacturer’s instructions. DNA sequencing was performed at Secugen S.L. (Madrid, Spain) on 3730 XL DNA Analyzer (Applied Biosystems) using BigDye^®^ Terminator v3.1 reagents (Thermo Fisher Scientific). Oligonucleotides used for sequencing *Ccα6* codifying sequence were: RnACh6-535 (5′-CCAAATCCAACTGATTTCCATCGT-3′), FnACh6-996 (5′-TAAATCCGTTTTCCTGCAATGGCT-3′), and RnACh6-1175 (5′-ATCGTATGCCGGAAATCATCATCG-3′).

### Genomic DNA extraction, PCR and sequencing of *Ccα6* exon 3a and exon 3b

Genomic DNA extraction from thorax and abdomen of single individuals was carried out through tissue homogenization and DNA isolation using DNeasy Blood & Tissue kit (Qiagen), following manufacturer’s instructions. DNA was quantified and 100–200 ng were used as template for PCR in a volume of 10 μl, along with: 0.4 μM of forward and reverse oligonucleotides, 0.5 U AmpliTaq Gold (Applied Biosystems), 10x PCR buffer II, 2 mM MgCl_2_, and 0.64 mM dNTPs. Oligonucleotides used for exon 3a amplification were FnACh6ex3a-intron (5′-ACGAAGGCGAAATAAGTTTCAAGT-3′) and RnACh6ex3a-intron (5′-GCTGCGTGAAAACCATTGAAATCG-3′). Oligonucleotides used for exon 3b amplification were FnACh6ex3b-intron (5′-CTTTTTATGTTCAATGTCTTCTGC-3′) and RnACh6ex3b-intron (5′-TTTGGTGCCACTTCGTATGCATGA-3′). For both amplifications cycling conditions consisted on an initial denaturation step at 95 °C for 5 min, followed by 40 cycles of 95 °C for 30 s, 57 °C for 30 s, and 72 °C for 25 s, and a final step of 72 °C for 7 min to fully extend all PCR products. Sequencing was performed as previously described, directly from PCR products, using FnACh6ex3a-intron or RnACh6ex3b-intron oligonucleotides.

### PCR-RFLP for K352* detection on exon 10

The presence/absence of K352* mutation was detected through a PCR-RFLP method, taking advantage of the generation of an MseI restriction site in the presence of this mutation. Exon 10 was amplified in a volume of 10 μl using 100–200 ng of genomic DNA as template, 0.4 μM of FnACh6-996 (5′-TAAATCCGTTTTCCTGCAATGGCT-3′) and RnACh6-1111 (5′-CCTTTAATTCCAATTCCTTCATGC-3′) oligonucleotides, 0.5 U AmpliTaq Gold (Applied Biosystems), 10x PCR buffer II, 2.5 mM MgCl_2_, and 0.64 mM dNTPs. PCR conditions were as follows: an initial denaturation step at 95 °C for 5 min; followed by 40 cycles of 95 °C for 30 s, 60 °C for 30 s, and 72 °C for 25 s; and a final step of 72 °C for 7 min to complete the extension. PCR products were then digested at 37 °C for 2 hours with 2 U of MseI restriction enzyme (New England BioLabs) and 10x CutSmart^®^ buffer (New England BioLabs). Digestion products were finally analysed by electrophoresis on a 2.5% agarose gel composed of a 1:1 (vol/vol) mix of conventional agarose D2 and low melting temperature agarose (NuSieve^TM^ GTG^TM^ Agarose, Lonza). Two superposed bands of 58 bp in the agarose gel indicate a homozygous genotype for the K352* mutation. A single non-digested band of 116 bp indicates that the individual do not carry the mutation. Finally, a heterozygous individual produce the non-digested band (116 bp) and the two digested bands (58 bp) (see Supplementary Information, Fig. S2A for a representative image).

### Multiplex PCR methods to detect AG > AT and Q68* mutations on exon 3a

A combination of multiplex PCRs was designed to detect the mutations observed in exon 3a (AG > AT and Q68*) from genomic DNA without the sequencing step. Multiplex PCR-1 to detect both mutations at the same time was performed in a volume of 10 μl. Oligonucleotides used in this multiplex were: 0.4 μM of a forward oligonucleotide specific for the alleles with the splicing site mutation AG > AT and the linked A200T mutation, including also a mismatch on 5′ region to confer more stability to the primer (FnACh6ex3a-splicing, 5′-ATTATTTATATGACGAAAAGATTC-3′)^39^; 0.4 μM of a reverse oligonucleotide specific for the alleles with the Q68* mutation, including a destabilizing mismatch on the third nucleotide close to the 3′ end to avoid unspecific annealing on wild-type sequences (RnACh6ex3a-Q68Stop, 5′-CCACGCATTTGTGGTCAGAATTTA−3′); and 0.1 μM of FnACh6ex3a-intron and RnACh6ex3a-intron oligonucleotides, common to all the alleles (Supplementary Information, Fig. S3). Other components required for the PCR were: 100–200 ng of genomic DNA, 0.5 U AmpliTaq Gold, 10x PCR buffer II, 2 mM MgCl_2_, and 0.64 mM dNTPs. Cycling conditions consisted on a denaturation step at 95 °C for 5 min, followed by 40 cycles of 95 °C for 30 s, 50 °C for 30 s, and 72 °C for 20 s, and a final step of 72 °C for 7 min to complete the extension. PCR products were finally analysed by electrophoresis on a 2.5% agarose gel composed of a 1:1 (vol/vol) mix of conventional agarose D2 and low melting temperature agarose. Multiplex PCR using these four oligonucleotides generated four different patterns of bands (Supplementary Information, Fig. S2B and S3) that allowed us to discriminate among: 1- homozygous genotypes without any of the two mutations (+/+) (320 bp band); 2- homozygous for the splicing mutation (AG > AT/AG > AT) and heterozygous with no mutations in one allele and the splicing mutation in the other (+/AG > AT) (two bands of 320 bp and 209 bp); 3- homozygous for the Q68* mutation (Q68*/Q68*) or heterozygous with no mutations in one allele and Q68* mutation in the other (+/Q68*) (two bands of 320 bp and 158 bp); and 4- heterozygous with the splicing mutation in one allele and Q68* mutation in the other (Q68*/AG > AT) (three bands of 320 bp, 209 bp and 158 bp). Thus, although this multiplex PCR allows the detection of the two mutations observed in exon 3a, it has the handicap that it cannot discriminate between the +/AG > AT and AG > AT/AG > AT genotypes and between the +/Q68* and Q68*/Q68* genotypes.

Differentiation between homozygous Q68*/Q68* and heterozygous +/Q68* individuals was carried out through multiplex PCR-2 (Supplementary Information, Fig. S2C). A new forward oligonucleotide was designed on the Q68 region, specific for the wild-type sequences including an extra destabilizing mismatch on the third nucleotide close to the 3′ end to avoid unspecific annealing (FnACh6ex3a-wtQ68, 5′-ATTTTTTATAGGACGAAAAGAGTC−3′) (Supplementary Information, Fig. S3). It was used together with the reverse oligonucleotide specific for the alleles with the Q68* (RnACh6ex3a-Q68Stop) and the two intronic oligonucleotides common to all alleles (FnACh6ex3a-intron and RnACh6ex3a-intron), to allow the amplification. PCR components for 10 μl of multiplex PCR-2 were as follows: 100–200 ng of genomic DNA, 0.1 μM of FnACh6ex3a-intron and RnACh6ex3a-intron oligonucleotides, 0.2 μM of FnACh6ex3a-wtQ68, 0.4 μM of RnACh6ex3a-Q68Stop, 0.5 U AmpliTaq Gold, 10x PCR buffer II, 2 mM MgCl_2_, and 0.64 mM dNTPs. Amplification conditions consisted on a denaturation step at 95 °C for 5 min, followed by 40 cycles of 95 °C for 30 s, 50 °C for 30 s, and 72 °C for 20 s, and a final step of 72 °C for 7 min to complete the extension. PCR products were analysed by electrophoresis on a 2.5% agarose gel composed of a 1:1 (vol/vol) mix of conventional agarose D2 and low melting temperature agarose. The different pattern of bands depending on the genotype allowed to differentiate among: 1- homozygous genotypes without the Q68* mutation (+/+) (two bands of 320 bp and 209 bp); 2- heterozygous with no mutations in one allele and Q68* mutation in the other (+/Q68*) (three bands of 320 bp, 209 bp and 158 bp); and 3- homozygous with Q68* mutation in both alleles (two bands of 320 bp and 158 bp) (Supplementary Information, Fig. S2C).

Finally, multiplex PCR-3 was designed to distinguish between +/AG > AT and AG > AT/AG > AT individuals (Supplementary Information, Fig. S2D). A reverse oligonucleotide specific for the wild-type alleles was designed on the splicing domain region (RnACh6ex3a-wtAG, 5′-GTCAGAATCTGATTCTTTTCGTCC−3′) (Supplementary Information, Fig. S3). It was used together with the forward oligonucleotide specific for the alleles with the AG > AT mutation (FnACh6ex3a-splicing) and the two intronic oligonucleotides common to all alleles (FnACh6ex3a-intron and RnACh6ex3a-intron), to allow the amplification. PCR was performed in a volume of 10 μl containing: 100–200 ng of genomic DNA, 0.1 μM of FnACh6ex3a-intron and RnACh6ex3a-intron oligonucleotides, 0.4 μM of FnACh6ex3a-splicing, 0.1 μM of RnACh6ex3a-wtAG, 0.5 U AmpliTaq Gold, 10x PCR buffer II, 2 mM MgCl_2_, and 0.64 mM dNTPs. Cycling conditions consisted on a denaturation step at 95 °C for 5 min, followed by 40 cycles of 95 °C for 30 s, 55 °C for 30 s, and 72 °C for 20 s, and a final step of 72 °C for 7 min to complete the extension. PCR products were analysed by electrophoresis on a 2.5% agarose gel composed of a 1:1 (vol/vol) mix of conventional agarose and low melting temperature agarose. The pattern of bands obtained allowed to differentiate among: 1- homozygous genotypes without the AG > AT mutation (+/+) (two bands of 320 bp and 145 bp); 2- heterozygous with no mutations in one allele and AG > AT mutation in the other (+/AG > AT) (three bands of 320 bp, 209 bp and 145 bp); and 3- homozygous with AG > AT mutation in both alleles (two bands of 320 bp and 209 bp, but with slight unspecific annealing of RnACh6ex3a-wtAG oligonucleotide that shows a weak band of 145 bp) (Supplementary Information, Fig. S2D). An unspecific band of approximately 400 bp that does not interfere on the interpretation of the results was also visible in +/AG > AT and AG > AT/AG > AT genotypes (Supplementary Information, Fig. S2D).

## Results

### Spanish field populations show high susceptibility to spinosad

We first assessed the spinosad susceptibility of twelve *C*. *capitata* field populations collected in Spain (Supplementary Information, Fig. S1 and Table S1) by concentration-response bioassays (Table 1) and by discriminating concentration assays (Supplementary Information, Table S2). We found that LC_50_ values, which estimate the concentration killing 50% of the individuals, were always far below the concentration of the spinosad-based insecticide recommended for *C*. *capitata* treatments in Spain (240 ppm, Spintor Cebo 0.024% p/v, Dow Agrosciences) (Table 1). In addition, we found that resistance ratios (RR) calculated for field populations in comparison to the susceptible laboratory control strain (C strain) were always below three-fold. Similarly, treatments with the discriminating concentration of 1 ppm of spinosad, which causes 88% mortality in the C strain, also caused mortality ≥77% in all field populations tested (Supplementary Information, Table S2). Thus, our initial screening demonstrated high susceptibility to spinosad in all medfly field populations analysed.

**Table 1:** Susceptibility to spinosad of field populations and a control laboratory strain (C) of *Ceratitis capitata*.

Population	Year	n^†^	slope ± S.E.	LC_50_^‡^ (95% FL)	χ^2^	df	RR (95% FL)^§^
Laboratory (C)	—	305	4.44 ± 1.01	0.58 (0.50–0.73)	12.2*	18	—
Xàbia (Alacant)	2007	244	2.38 ± 0.43	1.56 (0.94–2.19)	22.1*	18	2.69 (1.77–4.10)^♯^
Villalengua (Zaragoza)	2007	299	2.84 ± 0.38	0.62 (0.51–0.73)	13.5*	18	1.07 (0.82–1.40)
Castellserà (Lleida)	2009	359	3.48 ± 0.54	0.95 (0.83–1.06)	17.3*	22	1.63 (1.29–2.07)^♯^
Sagunt (València)	2009	246	12.3 ± 3.2	1.33 (1.22–1.41)	17.8*	18	2.28 (1.85–2.81)^♯^
	2010	336	4.98 ± 0.64	1.68 (1.56–1.82)	11.8*	22	2.89 (2.34–3.56)^♯^
	2015	199	2.56 ± 0.69	1.46 (1.12–1.75)	7.6*	10	2.50 (1.84–3.40)^♯^
Albal (València)	2015	172	2.21 ± 0.56	1.01 (0.57–1.33)	13.5*	12	1.73 (1.12–2.65)^♯^
Algarrobo Costa (Málaga)	2015	133	6.91 ± 1.22	0.96 (0.77–1.21)	18.3	8	1.65 (1.25–2.17)^♯^

^†^Number of flies considered in the Probit analysis (including non-treated).

^‡^Lethal concentration (LC_50_) in ppm of spinosad in the diet at 48 h. Feeding assays performed with Spintor Cebo in 2007 and with spinosad 88% technical grade insecticide since 2009.

^§^Resistance ratio (RR) = LC50 (field strain)*/*LC50 (C strain). The fiducial limits for RR were calculated according to Robertson and Preisler (1992). ^♯^RR is significant (P < 0.05) if the 95% FL does not include 1.

*Good fit of the data to the probit model (P > 0.05).

### The laboratory-selected strain JW-100 s is resistant to spinosad

In order to obtain a spinosad-resistant strain, we adapted one of the populations with higher LC_50_, Xàbia 2007, to laboratory conditions and selected the strain over successive generations with increasing concentrations of spinosad in the adult rearing diet (Table 2). It was not until generation F25 that we observed a moderate increase in the RR (5.79-fold). After generation F25, we observed that resistance levels increased rapidly, with RR reaching 321-fold at generation F29. We set the selection pressure at 100 ppm of spinosad in the diet from generation F30, but we could not perform feeding bioassays to calculate LC_50_ ratios after generation F36 because of the limited solubility of spinosad in water solutions (235 ppm at pH 7 and 290 ppm at pH 5 for spinosyn A, SPINOSAD Technical Bulletin, Dow AgroSciences LLC). Accordingly, we performed topical bioassays using acetone as a solvent from generation F35, after which we were able to achieve an RR of over 1000-fold at generation F45 compared to the susceptibility of the C strain. We observed similar levels of resistance until the last generation evaluated (F81). We named the laboratory-selected spinosad-resistant strain JW-100 s.

**Table 2:** Selection of resistance to spinosad to obtain the JW-100 s strain.

Gen.	SC^a^	Bioassay^b^	n^c^	slope ± S.E.	LC_50_^d^ (95% FL)	χ^2^	d.f.	RR (95% FL)^e^
F0^f^	—	feeding	244	2.38 ± 0.43	1.56 (0.94–2.19)	22.1*	18	2.69 (1.77–4.10)^♯^
F14	2.5	feeding	262	2.49 ± 0.38	0.62 (0.45–0.77)	11.4*	14	1.06 (0.76–1.50)
F21	4.0	feeding	250	4.20 ± 0.59	1.36 (1.21–1.57)	15.1*	14	2.35 (1.87–2.96)^♯^
F25	6.5	feeding	317	2.48 ± 0.35	3.37 (2.81–4.33)	20.4*	18	5.79 (4.38–7.66)^♯^
F29	10	feeding	329	1.95 ± 0.40	187 (152–262)	12.5*	18	321 (228–454)^♯^
F36	100	feeding	365	1.86 ± 0.29	157 (126–207)	28.0*	22	269 (202–359)^♯^
					**LD**_**50**_^**d**^ **(95% FL)**			
F35	100	topical	195	4.97 ± 0.87	5.34 (4.27–6.27)	13.1*	10	51.9 (32.9–82.1)^♯^
F40	100	topical	195	1.33 ± 0.30	39.4 (25.4–67.8)	4.6*	10	383 (196–749)^♯^
F45	100	topical	131	5.59 ± 0.85	159 (134–199)	8.1*	7	1550 (977–2457)^♯^
F48	100	topical	287	11.93 ± 2.78	116 (104–125)	17.7*	16	1125 (726–1743)^♯^
F81	100	topical	419	4.74 ± 0.61	185 (142–224)	83.7	22	1794 (1156–2785)^♯^

^a^Selection concentrations (SC) used in the selection process, in ppm of spinosad in the diet. The absence of treatment is indicated as “−”.

^b^Assays were performed with Spintor Cebo until F13. Spinosad 88% technical grade insecticide was used since F14.

^c^Number of flies considered in the Probit analysis (including non-treated).

^d^Lethal concentration (LC_50_) in ppm of spinosad in the diet for feeding bioassays at 48 h. Lethal dose (LD_50_) in µg/g of insect (fresh weight assuming an average weight of 10 mg) by topical application at 48 h (a 0.5 μL drop of insecticide solution in acetone or acetone alone was applied to the dorsal thorax of each fly by using an automatic microapplicator).

^e^Resistance ratio (RR) = [LC_50_ (selected strain)*/*LC_50_ (C strain, data from Table 1)] or [LD_50_ (selected strain)*/*LD_50_ (C strain, {n = 156; slope ± S.E. = 5.66 ± 1.78; LD50 (95% FL) = 0.12 (0.10–0.15); χ^2^ = 6.4*; d.f. = 7})] for feeding or topical bioassays, respectively. The fiducial limits for RR were calculated according to Robertson and Preisler (1992). ^♯^RR is significant (P < 0.05) if the 95% FL does not include 1.

^f^Field population collected in Xàbia in 2007, data from Table 1.

*Good fit of the data to the probit model (P > 0.05).

### The resistance in JW-100 s strain is not reverted by inhibitors of detoxification enzymes and does not confer cross-resistance to other insecticides

To decipher the molecular mechanisms associated with spinosad resistance in JW-100 s, we tested the role of detoxification enzymes using the synergists PBO (inhibitor of cytochrome P450 s), DEF (esterase inhibitor) and DEM (inhibitor of glutathione S-transferases) (Table 3). Topical treatment with PBO or DEF on JW-100 s produced a slight reduction on LD_50_, with a synergistic ratio (SR) of 1.91 and 1.73, respectively, whilst treatment with DEM did not influence the susceptibility of the strain (SR = 0.96). These results suggested a minor contribution of detoxification enzymes to the spinosad resistance found in the JW-100 s strain.

**Table 3:** Effect of synergists on the resistance of JW-100 s to spinosad by topical application.

Synergist^†^	n^‡^	slope ± S.E.	LD_50_^§^ (95% CL)	χ^2^	d.f.	SR (95% FL)^¶^
PBO−	272	2.84 ± 0.52	98.2 (76.6–119)	13.5*	16	
PBO+	260	2.64 ± 0.52	51.4 (36.0–66.1)	8.8*	16	1.91 (1.29–2.82)^#^
DEM−	186	2.59 ± 0.55	265 (217–360)	6.9*	10	
DEM+	194	1.99 ± 0.52	276 (216–447)	3.0*	10	0.96 (0.60–1.53)
DEF−	390	3.99 ± 1.30	298 (218–336)	13.3*	22	
DEF+	382	2.66 ± 0.38	172 (137–204)	21.1*	22	1.73 (1.28–2.34)^#^

^†^Synergists: 0.5 μg PBO, 1 μg DEF and 1 μg DEM diluted in 0.5 μl acetone and topically applied (acetone was used as control without synergist). After 2 h the flies were treated with spinosad.

^‡^Number of flies considered in the Probit analysis (including non-treated).

^§^Lethal dose (LD_50_) in µg/g of insect (fresh weight assuming an average weight of 10 mg) by topical application at 48 h (a 0.5 μL drop of insecticide solution in acetone or acetone alone was applied to the dorsal thorax of each fly by using an automatic microapplicator). Spinosad 88%, technical grade insecticide.

^¶^Synergistic ratio (SR) = LD_50_ (synergist −)*/*LD_50_ (synergist+). The fiducial limits for SR were calculated according to Robertson and Preisler (1992). ^♯^SR is significant (P < 0.05) if the 95% FL does not include 1.

*Good fit of the data to the probit model (P > 0.05).

To further explore the mechanisms associated with resistance, we next tested the cross-resistance of the JW-100 s strain to the insecticides imidacloprid, fipronil, malathion and lambda-cyhalotrin (Table 4). Feeding treatments with discriminating concentrations of malathion and lambda-cyhalotrin revealed a high susceptibility of the JW-100 s strain to these two insecticides. On the contrary, we observed high tolerance to imidacloprid and fipronil after feeding treatments (RR of 6.5- and 17.5-fold when compared to the C strain, respectively). However, we did not observe this tolerance after topical treatments, suggesting that it could be related to a detoxification mechanism in the digestive tract, bypassed under topical application. These results demonstrated that the main mechanism responsible for the resistance to spinosad in JW-100 s did not confer cross-resistance to the insecticides imidacloprid, fipronil, malathion or lambda-cyhalotrin.

**Table 4:** Cross-resistance of JW-100 s strain to imidacloprid, fipronil, malathion and lambda-cyhalothrin.

Insecticide	Strain	n^†^	slope ± S.E.	LC_50_^‡^ (95% CL)	χ^2^	d.f.	RR (95% FL)^§^
**feeding**							
Imidacloprid	C	317	1.24 ± 0.16	165 (117–236)	18.9*	22	
	JW-100 s	411	0.61 ± 0.14	1062 (550–36732)	27.6*	22	6.5 (2.6–15.9)^#^
Fipronil	C	262	3.25 ± 0.44	21.8 (16.9–27.9)	21.6*	14	
	JW-100 s	298	2.75 ± 0.51	382 (231–478)	27.9	14	17.5 (12.8–23.9)^#^
Malathion	C			63% M at 30 ppm & 100% M at 300 ppm			
	JW-100 s			42% M at 30 ppm & 98% M at 300 ppm			
Lambda-cyhalothrin	C			68% M at 30 ppm & 87% M at 300 ppm			
	JW-100 s			48% M at 30 ppm & 75% M at 300 ppm			
				**LD**_**50**_^**‡**^ **(95% FL)**			
**topical**							
Imidacloprid	C	272	1.13 ± 0.25	17.5 (10.8–41.2)	18.9*	16	
	JW-100 s	273	1.00 ± 0.16	15.3 (9.2–26.0)	20.5*	16	0.87 (0.42–1.83)
Fipronil	C	351	1.70 ± 0.25	2.23 (1.65–2.89)	21.6*	22	
	JW-100 s	274	1.49 ± 0.25	1.26 (0.76–1.88)	24.8*	16	0.56 (0.36–0.89)^#^

^†^Number of flies considered in the Probit analysis (including non-treated).

^‡^Lethal concentration (LC_50_) in ppm of insecticide in the diet for the feeding bioassays at 48 h. For malathion and lambda-cyhalothrin, % mortality (M) at two discriminant concentrations were tested. Lethal dose (LD_50_) in µg/g of insect (fresh weight assuming an average weight of 10 mg) by topical application at 48 h (a 0.5 μL drop of insecticide solution in acetone or acetone alone was applied to the dorsal thorax of each fly by using an automatic microapplicator).

^§^Resistance ratio (RR) = LC_50_ (JW-100 s strain)*/*LC_50_ (C strain) or LD_50_ (JW-100 s strain)*/*LD_50_ (C strain). The fiducial limits for RR were calculated according to Robertson and Preisler (1992). ^♯^RR is significant (P < 0.05) if the 95% FL does not include 1.

*Good fit of the data to the probit model (P > 0.05).

### The JW-100 s strain contains diverse mutations in the *Ccα6* gene

Next, we examined the proposed primary target site of spinosad, the α6 subunit of the nAChR^6,22^, encoded by the *Ccα6* gene^5^ [accession no. MK251468 (*Ccα6 3a*–*8a*); MK251469 (*Ccα6 3a*–*8b*); MK251470 (*Ccα6 3b*–*8a*); and MK251471 (*Ccα6 3b*–*8b*)]. We obtained the *Ccα6* coding sequence by RT-PCR and direct sequencing from five non-treated individuals of the spinosad resistant strain JW-100 s (F81). We observed double traces in the chromatograms compatible with the expression of alternative exons 3a/3b and 8a/8b, but not 8c. We also observed conserved A-to-I RNA editing sites 4, 5, 6 and 7^9^. Interestingly, we identified two homozygous point mutations generating premature stop codons in all of the five individuals of the JW-100 s strain analysed. One point mutation was located among the double peaks corresponding to the simultaneous sequencing of exons 3a/3b (Supplementary Information, Fig. S4A). We determined the precise location of this mutation at exon 3a (nt: C202T; aa: Q68*) by PCR amplification and direct sequencing of exon 3a and exon 3b from genomic DNA (Supplementary Information, Fig. S4A). The second point mutation corresponded to the coding sequence of exon 10 (nt: A1054T; aa: K352*) (Supplementary Information, Fig. S4B). We next analysed the presence of these point mutations in a larger cohort of individuals (n = 48) from generation F85 of the JW-100 s strain, using direct sequencing, PCR-RFLP and multiplex PCR on genomic DNA. Our analysis demonstrated the existence of two alleles: *Ccα6*^3aQ68–K352*^ carrying the two premature stop codon mutations at exon 3a and exon 10; and *Ccα6*^3aQ68*^ carrying only the stop codon mutation at exon 3a (Supplementary Information, Table S3). Remarkably, 92% of the non-treated individuals of the JW-100 s strain analysed from F85 were homozygous for *Ccα6*^3aQ68*-K352*^, and the rest were heterozygous for both *Ccα6*^3aQ68*-K352*^ and *Ccα6*^3aQ68*^ alleles (Table 5). We observed similar results among individuals from generation F85 that survived a treatment of 100 ppm of spinosad in the diet. On the other hand, we did not observe any of these *Ccα6* mutant alleles in three different laboratory strains highly susceptible to spinosad (C, W-4 km and W-1 kλ) ^31,33^ (Table 5). These results suggested that *Ccα6*^3aQ68*-K352*^ and *Ccα6*^3aQ68*^ alleles were associated with spinosad resistance in JW-100 s laboratory strain.

**Table 5:** Genotype frequency in different generations of JW-100 s strain, and in C, W-4 km and W-1 kλ strains. Individuals analysed are non-treated or survivors under the indicated spinosad concentrations/doses.

Strain	Gen.	Spinosad treatment^†^	n^‡^	Genotype frequency							
				+/+	+/*Ccα6*^3aQ68*^	*Ccα6* ^3aQ68*^ */Ccα6* ^3aQ68*^	+/*Ccα6*^*3aAG*>*AT*^	*Ccα6* ^*3aAG*> *AT*^ */Ccα6* ^*3aAG*> *AT*^	*Ccα6* ^3aQ68*^ */Ccα6* ^*3aAG*> *AT*^	*Ccα6* ^3aQ68*^ */Ccα6* ^3aQ68*-K352*^	*Ccα6* ^3aQ68*-K352*^ */Ccα6* ^3aQ68*-K352*^
JW-100 s	F0	nt	5	1	—	—	—	—	—	—	—
		1–5^§^ ppm	37	1	—	—	—	—	—	—	—
	F25	nt	30	0.07	0.33	0.07	0.17	0.07	0.3	—	—
		6 ppm	20	—	—	0.3	0.1	0.15	0.45	—	—
	F29	nt	30	—	—	0.3	—	0.2	0.5	—	—
		240 ppm	20	—	—	0.65	—	0.05	0.3	—	—
	F48	nt	24	—	—	—	—	—	—	0.042	0.958
		100 μg/g	24	—	—	—	—	—	—	—	1
	F85	nt	48	—	—	—	—	—	—	0.083	0.917
		100 ppm	15	—	—	—	—	—	—	0.067	0.933
Control	F130	nt	10	1	—	—	—	—	—	—	—
W-4 Km	F107	nt	10	1	—	—	—	—	—	—	—
W-1Kλ	F62	nt	10	1	—	—	—	—	—	—	—

^†^Spinosad was administered by feeding (concentration in ppm) on F0, F25, F29 and F85, and by topical application (dose in μg/g) on F48. No treatment (nt).

^‡^Number of flies genotyped.

^§^Includes 26 survivors at 1 ppm, 8 survivors at 3 ppm and 3 survivors at 5 ppm.

“+” refers to the alleles bearing none of the mutations described (AG > AT, Q68* or K352*).

“−” means that the frequency detected for a specific genotype was 0.

To further correlate the presence of the described alleles and the resistance to spinosad in JW-100 s strain, we recorded the frequency of the two alleles in non-treated individuals and in individuals surviving spinosad exposure at generations F0, F25, F29 and F48. Interestingly, we identified an additional new allele in generation F25 from sequencing the genomic region of exon 3a. This new allele, *Ccα6*^*3aAG*>*AT*^, carries a mutation at the 5′ canonical splicing dinucleotide of exon 3a, changing the AG splice site to AT (AG > AT), together with another point mutation at the coding region of exon 3a (nt: A200T) (Supplementary Information, Fig. S4C). This allele does not carry the mutations 3aQ68* or K352* (Supplementary Information, Table S3). Chromatograms obtained from individuals homozygous for the *Ccα6*^3aQ68*^ allele demonstrated that this allele produced isoforms containing the exon 3b. On the other hand, chromatograms from individuals homozygous for the *Ccα6*^*3aAG*>*AT*^ allele demonstrated that this allele produced full-length 3b transcripts, but also produced incomplete 3a transcripts by skipping exon 3a and joining exons 2 and 4 without altering the coding frame of the gene.

We next used PCR-RFLP and multiplex PCR methods to detect the presence of the three mutations and thus identify the specific genotype of each individual (Supplementary Information, Table S3). We called alleles that did not bear any of the described mutations ‘+’ alleles. The frequencies of genotypes and alleles in each generation are shown in Table 5 and Supplementary Information, Table S4, respectively. We did not find any of the described mutations among the F0 individuals that generated the JW-100 s strain, including both non-treated individuals and the survivors at different spinosad concentrations. However, in generation F25, when we observed a small increase on the level of resistance (Table 2), we detected *Ccα6*^*3aAG*>*AT*^ and *Ccα6*^3aQ68*^ alleles with a frequency of 0.3 and 0.38, respectively, in non-treated individuals (Supplementary Information, Table S4). Surprisingly, we did not detect the allele *Ccα6*^3aQ68*-K352*^ in this generation. Among the genotyped individuals that survived to a concentration of 6 ppm of spinosad in the same generation (F25), a low frequency (0.1) had a wild type allele (+/*Ccα6*^*3aAG*>*AT*^), while all the rest had both mutated alleles (*Ccα6*^*3aAG*>*AT*^/*Ccα6*^*3aAG*>*AT*^, *Ccα6*^3aQ68*^/*Ccα6*^3aQ68*^ or *Ccα6*^*3aAG*>*AT*^/*Ccα6*^3aQ68*^) (Table 5). Four generations later (F29), when the resistance of JW-100 s increased to 321-fold, the frequency of *Ccα6*^*3aAG*>*AT*^ and *Ccα6*^3aQ68*^ alleles among the non-treated individuals increased (0.45 and 0.55, respectively), while + alleles were not detected (Supplementary Information, Table S4). Among the survivors at a high spinosad dose (240 ppm), the frequency of *Ccα6*^3aQ68*^ allele (0.8) was higher than the *Ccα6*^*3aAG*>*AT*^ allele (0.2), and the *Ccα6*^*3aAG*>*AT*^/*Ccα6*^*3aAG*>*AT*^ genotype was observed only in one individual among 30 analysed (Table 5 and Supplementary Information, Table S4). Finally, in generation F48 we found the *Ccα6*^3aQ68*-K352*^ allele at a high frequency (0.98), whilst the frequency of *Ccα6*^3aQ68*^ allele diminished (0.02) and the *Ccα6*^*3aAG*>*AT*^ allele was not detected (Table 5 and Supplementary Information, Table S4). Altogether, these results further demonstrated that alleles *Ccα6*^3aQ68*-K352*^, *Ccα6*^3aQ68*^ and *Ccα6*^*3aAG*>*AT*^ were associated with spinosad resistance. It is worth noting that the three mutant alleles produced transcripts coding for truncated *Ccα6* isoforms. Interestingly, in *Ccα6*^3aQ68*^ and *Ccα6*^*3aAG*>*AT*^ alleles, this truncation would affect only the isoforms containing the exon 3a (see discussion and Fig. 1). Moreover, the predominance of the *Ccα6*^3aQ68*-K352*^ allele detected from generation F48 of the selection process suggested that this allele could be more advantageous than *Ccα6*^3aQ68*^ and *Ccα6*^*3aAG*>*AT*^ alleles.

### Spinosad resistance in JW-100 s is completely recessive and linked to *Ccα6*^3aQ68*-K352*^ and *Ccα6*^3aQ68*^ alleles

Finally, we studied the inheritance of spinosad resistance by performing reciprocal crosses of JW-100 s individuals from generation F83 with individuals of the susceptible C strain (Table 6). We confirmed that all individuals at F1 were heterozygous (+/*Ccα6*^3aQ68*-K352*^ or +/*Ccα6*^3aQ68*^) (Table 7). The LC_50_ values estimated for F1 descendants (0.75 and 0.78 ppm, Table 6) were only slightly higher than the LC_50_ value estimated for the control strain (0.58 ppm, Table 1), much smaller than that calculated at F36 for JW-100 s by feeding bioassays (157 ppm, Table 2). Moreover, the LC_50_ values did not differ between the two reciprocal crosses, suggesting an autosomal rather than X-linked inheritance pattern. The resulting dominance values (D_LC_) calculated in relation to this LC_50_ were close to 0 (0.05 and 0.06, Table 6). These results indicated that spinosad resistance was inherited as an autosomal and almost completely recessive trait.

**Table 6:** Reciprocal crosses between JW-100 s resistant strain and C susceptible strain.

		n^†^	slope ± S.E.	LC_50_^‡^ (95% CL)	χ^2^	d.f.	RR (95% FL)^§^	D_LC_^¶^
F1 crosses	♀JW-100 s x ♂C	240	7.96 ± 1.13	0.75 (0.68–0.81)	10.1*	10	1.29 (1.04–1.59)^#^	0.05
	♀C x ♂JW-100 s	240	4.37 ± 0.82	0.78 (0.68–0.91)	12.3*	10	1.34 (1.06–1.69)^#^	0.06
F2 crosses	♀JW-100 s x ♂C	420		91% M at 10 ppm				
	♀C x ♂JW-100 s	417		90% M at 10 ppm				

^†^Number of flies considered in the Probit analysis (including non-treated).

^‡^Lethal concentration (LC_50_) in ppm of insecticide (Spinosad 88%, technical grade) in the diet at 48 h.

^§^Resistance ratio (RR) = LC50 (JW-100 s strain or F1 crosses)*/*LC50 (C strain). The fiducial limits for RR were calculated according to Robertson and Preisler (1992). ^#^RR is significant (P < 0.05) if the 95% FL does not include 1.

^¶^Dominance ^(^D_LC_) = {[(2 log LC50 F1 −log LC50 C −log LC50 JW-100 s)*/*(log LC50 JW-100 s −log LC50 C)]+1}*/*2. Values range between 0 for completely recessive and 1 for completely dominant. LC_50_ for C from Table 1 and for JW-100 s from Table 2 (data corresponding to F36, the last generation in which LC50 was estimated).

*Good fit of the data to the probit model (P > 0.05).

**Table 7:** Genotype frequency on F1 and F2 individuals from JW-100 s x C reciprocal crosses and their resistance to spinosad.

	Crosses	Spinosad treatment	Phenotype	n^†^	Genotype frequency					
					+/+	+/*Ccα6*^3aQ68*^	+/*Ccα6*^3aQ68*-K352*^	*Ccα6* ^3aQ68*^ */Ccα6* ^3aQ68*-K352*^	*Ccα6* ^K352*^ */Ccα6* ^3aQ68*-K352*^	*Ccα6* ^3aQ68*-K352*^ */Ccα6* ^3aQ68*-K352*^
F1	♀JW-100 s x ♂C	0 ppm	alive	4	—	—	1	—	—	—
		0.8 ppm	alive	4	—	0.25	0.75	—	—	—
		0.8 ppm	dead	4	—	—	1	—	—	—
	♀C x ♂JW-100 s	0 ppm	alive	4	—	—	1	—	—	—
		0.8 ppm	alive	4	—	—	1	—	—	—
		0.8 ppm	dead	4	—	0.25	0.75	—	—	—
F2	♀JW-100 s x ♂C	10 ppm	alive	36	—	—	—	0.06	0.03	0.92
		10 ppm	dead	36	0.33	—	0.67	—	—	—
	♀C x ♂JW-100 s	10 ppm	alive	40	—	—	—	0.13	0.03	0.85
		10 ppm	dead	24	0.25	0.04	0.71	—	—	—

^†^Number of flies genotyped.

In order to analyse the linkage between spinosad resistance and *Ccα6*^3aQ68*-K352*^ and *Ccα6*^3aQ68*^ alleles, we next obtained F2 offspring from the reciprocal crosses, and we treated these individuals with a discriminating concentration of spinosad (10 ppm) causing approximately 90% mortality (Table 6). We genotyped all surviving individuals and a number of dead individuals from this treatment (Table 7). Notably, none of the 76 survivors carried the + allele, with a majority homozygous for the *Ccα6*^3aQ68*-K352*^ allele (67 individuals, frequency of 0.88), and a minority heterozygous for the two mutant alleles *Ccα6*^3aQ68*^ and *Ccα6*^3aQ68*-K352*^ (7 individuals, frequency of 0.09). Surprisingly, two individuals (frequency of 0.03) among the survivors were heterozygous for the mutation 3aQ68* and homozygous for the mutation K352* (as verified by sequencing genomic DNA), thus carrying the allele *Ccα6*^3aQ68*-K352^ in combination with an allele not previously detected, *Ccα6*^K352*^. On the other hand, all dead individuals genotyped from the F2 had at least one + allele, either in heterozygosis (42 individuals) or in homozygosis (18 individuals) (Table 7). Altogether, these results confirmed the linkage between resistance to spinosad and the *Ccα6*^3aQ68*^ and *Ccα6*^3aQ68*-K352*^ alleles in the JW-100 s strain, as well as the recessive character of the mutant alleles.

## Discussion

In a number of insect species, high levels of spinosad resistance have been associated with alterations in the *α6* gene affecting all the *α6* isoforms. Studies on *D*. *melanogaster* have found that deletion of the nAChR *α6* subunit gene, point mutations generating premature stop codons, or other mutations next to the Cys-loop motif result in resistance to spinosad^6,7,22,23^. Resistance to spinosad has also been associated in *P*. *xylostella* and *B*. *dorsalis* with truncated *α6* transcripts due to mis-splicings, insertions or deletions^24,25,30^; in *P*. *xylostella* with a 3-residue deletion in the TM4 domain^26^; in *T*. *absoluta* with exon 3 skipping in *α6* transcripts^27^; and in *F*. *occidentalis*, *T*. *absoluta* and *T*. *palmi* with a point mutation G275E at a transmembrane domain^28,29,40^. Here, we demonstrate that the resistance to spinosad selected in the JW-100 s strain of medfly is associated with three different mutant alleles of the nAChR *α6* subunit gene. The *Ccα6*^3aQ68*-K352*^ allele contains the mutations 3aQ68* and K352*, and would lead to isoforms truncated at exon 3a, and to 3b isoforms truncated at exon 10 (Fig. 1B). Thus, none of the *Ccα6* transcripts from homozygous individuals for the *Ccα6*^3aQ68*-K352*^ allele could generate complete Ccα6 protein products. On the other hand, the *Ccα6*^3aQ68*^ allele contains the mutation 3aQ68* that would affect only the isoforms containing exon 3a while generating wild-type full-length Ccα6 isoforms with exon 3b (Fig. 1C). Similarly, the *Ccα6*^*3aAG*>*AT*^ allele, which contains the AG > AT mutation present in the 5′ splicing site of exon 3a, would produce full-length 3b transcripts and incomplete transcripts that skip exon 3a and join exons 2 and 4 (Fig. 1D). Hence, our results suggest that the absence of the isoforms containing exon 3a could be enough to confer resistance, despite the expression of full-length 3b isoforms.

The specific involvement of α6 isoforms in spinosad susceptibility has previously been analysed in *D*. *melanogaster*^7,8^. Taking advantage of the Gal4-UAS system, Perry *et al*. (2015)^8^ demonstrated that the expression of any of the four different *Dmα6* isoforms (3a8a, 3a8b, 3b8a or 3b8b) is sufficient to restore spinosad susceptibility in an *α6-*deficient strain of *D*. *melanogaster*. However, the rescue of resistance by expressing a single *Dmα6* transcript using the Gal4-UAS system might be influenced by the particularities of the system, which usually confers a higher expression level than that in basal wild-type conditions. Moreover, the authors observed that leaky expression of the parental UAS constructs in the absence of Gal4 driver, particularly the UAS constructs containing 3a isoforms, is sufficient to partially rescue the susceptibility to spinosad in an *α6-*deficient strain. They propose that some isoforms could be “more responsive to spinosad or better able to form functional receptors than others”^8^. This hypothesis is in concordance with our results that suggest that removal of 3a isoforms could be sufficient to confer resistance to spinosad in the medfly. On the other hand, an alternative explanation is that the alteration of the expression of one isoform in medfly may interfere either with the expression of other isoforms or with the correct assembly of nAChR receptors targeted by spinosad. Finally, we cannot completely discount that other mutations that escaped the limits of our analysis, for example mutations in non-encoding regions, could affect the transcript stability or the translation of full-length wild-type 3b isoforms in *Ccα6*^3aQ68*^ and *Ccα6*^*3aAG*>*AT*^ alleles. Thus, further experiments generating mutations affecting specific isoforms would be required to confirm that the alteration of only some *α6* isoforms is sufficient to achieve resistance in the medfly.

The frequency of the different alleles observed in the JW-100 s population changed during the selection process. Interestingly, *Ccα6*^*3aAG*>*AT*^ and *Ccα6*^3aQ68*^ alleles were the most frequent when the selection pressure was relatively low, but they were almost completely substituted by *Ccα6*^3aQ68*-K352^ when the concentration of spinosad was raised to 100 ppm (see Tables 2 and 5). These changes may be explained by an unbalanced contribution of the different alleles to resistance and/or by the existence of an undetermined fitness cost associated with the different mutations. The unbalanced contribution to resistance between the *Ccα6*^*3aAG*>*AT*^ and *Ccα6*^3aQ68*^ alleles could explain the higher frequency of individuals carrying the allele *Ccα6*^3aQ68*^ in survivors to 240 ppm at generation F29 (Table 5). However, the decreased frequency of this allele detected in generation F48 may be indicative of an undetermined biological cost that could have influenced the selection in favour to *Ccα6*^3aQ68*-K352*^. The fitness cost associated with mutations in the *α6* gene has not been elucidated in depth in insect species yet. This gene is not essential for survival, as demonstrated in knockout strains of *D*. *melanogaster* that show no evidences of fitness disadvantage^6^. Moreover, quantification of biological parameters (development, fecundity, fertility and life span) in spinosad resistant strains of *H*. *virescens*^41^, *P*. *xylostella*^42^ and *H*. *armigera*^43^ only showed slight reductions in fitness, and no negative effect on fitness were reported for a *F*. *occidentalis* resistant strain^44^. However, reversion of resistance concomitantly with the relaxation of spinosad selection pressure, an indicator of biological cost, has been reported for *M*. *domestica*^45^, *L*. *trifolii*^12^, *S*. *litura*^46^, *P*. *xylostella*^42^, *T*. *absoluta*^47^ and *F*. *occidentalis*^48^. In mammals, diverse nAChRs are present in multiple tissues and participate in different functions, including learning and other complex behavioural traits^49^. Thus, we cannot discount the possibility that the loss of function of truncated *α6* transcripts may affect some behavioural pattern in resistant individuals, making them less competitive than wild-type individuals.

None of the mutations associated with spinosad resistance in this report were found in the analysis of the F0 individuals directly collected in the field. These results could be explained by a low frequency of the resistant alleles in the field. However, it is possible that mutations could have spontaneously occurred along the selection process in the laboratory. In addition, a distinct unknown mechanism may have contributed to spinosad tolerance at the first generations of selection. This mechanism could be responsible for the cross-resistance to imidacloprid and fipronil that was observed in feeding bioassays but not in topical treatments. Moreover, it could also account for the slight but significant effect of the synergists PBO and DEF on spinosad resistance. Remarkably, the malathion resistant medfly strain W-4 Km, crossed with the population collected in Xàbia at the beginning of the selection process (see material and methods section), shows a low but significant cross-resistance to spinosad and lufenuron that could be attributed to a detoxification mechanism^35^. Reports on low levels of spinosad resistance mediated by suspected detoxification are abundant in the scientific literature^21^.

There is limited sustainability of the use of insecticides in bait sprays for the control of medfly, according to the recent reports on the resistance to malathion and lambda-cyhalothrin detected in Spanish field populations^31,33^. Other insecticides are currently registered for bait treatments against medfly in citrus groves. Deltamethrin is used in lure and kill traps, but resistance to lambda-cyhalothrin may confer cross-resistance to this insecticide as demonstrated in a laboratory strain^33^. Our analysis of the susceptibility to spinosad in populations collected at different locations and in different years indicates that resistance has not evolved in Spanish field populations (Table 1 and Supplementary Information, Table S2), and as far as we know no previous reports exist on resistance to this insecticide in medfly field populations. We have demonstrated that the *Ccα6*^3aQ68*-K352^ allele is almost completely recessive. In addition, other resistant alleles (*Ccα6*^*3aAG*>*AT*^, *Ccα6*^3aQ68*^ and *Ccα6*^K352*^) are likely to be recessive, as all individuals surviving spinosad carried either one of these resistance alleles in homozygosis or two of them in heterozygosis (see Tables 5 and 7). Thus, the presence of at least one copy of the wildtype nAChR *α6* gene is sufficient to avoid spinosad resistance. Accordingly, spinosad resistance in *D*. *melanogaster* α6 null strains^6^ and in the Pearl-Sel strain of *P*. *xylostella* that produces mis-spliced α6 transcripts^13,24^ are also recessive. The recessive nature of the resistance alleles, together with a potential fitness cost for resistance, may be an impediment for their selection in the field. However, resistance to spinosad caused by residue substitutions at the conserved Cys-loop of the subunit may be incompletely dominant, as is the case for the P146S mutation recently reported in *D*. *melanogaster*^7^. Moreover, our study highlights the plasticity of the *Ccα6* gene to acquire and support mutations. This plasticity could favour resistance evolution in the field, since different resistance alleles at low frequency could be combined in heterozygous individuals that would survive spinosad treatment. This, together with the successful laboratory selection of Xàbia field population to obtain the resistant strain JW-100 s, highlights that resistance could evolve if medfly management relies extensively on the use of this insecticide. This scenario underscores the need to implement resistance management strategies to counteract the selection of resistance in field populations of medfly.

### Ethical approval

This article does not contain any studies with human participants or animals performed by any of the authors.

## Supplementary information

Supplementary Information

## Data Availability

The datasets generated during and/or analysed during the current study are available from the corresponding author on reasonable request.
