# Supplementary material for: Multiple mutations in the nicotinic acetylcholine receptor Ccα6 gene associated with resistance to spinosad in medfly
Source: Sci Rep. 2019 Feb 27;9:2961. doi: 10.1038/s41598-019-38681-w (PMC6393475; doi:10.1038/s41598-019-38681-w)

Multiple mutations in the nicotinic acetylcholine receptor *Ccα6* gene associated with resistance to spinosad in medfly

Enric Ureña[1,2], Ana Guillem-Amat[1], Francisco Couso-Ferrer[1], Beatriz Beroiz[1], Nathalia Perera[1], Elena López-Errasquín[1], Pedro Castañera[1], Félix Ortego[1], Pedro Hernández-Crespo[1,*]

[1] Departamento de Biotecnología Microbiana y de Plantas, Centro de Investigaciones Biológicas, CSIC, Madrid, Spain

[2] Present Address: Institute of Healthy Ageing, Department of Genetics, Evolution and Environment, University College London, Gower St, London WC1E 6BT, UK

* corresponding author

email: pedro@cib.csic.es

telephone: +34 918273112

ORCID: https://orcid.org/0000-0001-9350-3640

Table S1. *Ceratitis capitata* field populations used for the analysis of spinosad resistance

Population	Year	Host	Field Treatments
Xàbia (Alacant)	2007	Citrus	No treatment
Villalengua (Zaragoza)	2007	Peach	No treatment in 2007
La Vila Joiosa (Alacant)	2007	Citrus	No treatment in 2007
Almuñécar (Granada)	2007	Medlar	No treatment in 2007
Valle de Río Verde (Granada)	2007	Medlar	No treatment in 2007
Alcanar (Tarragona)	2007	Citrus	Malathion in 2005-2006 and malathion, spinosad and lambda-cyhalothrin in 2007
Muro (Mallorca)	2007	Citrus	Fenthion in 2005-2006 and methyl-chlorpyrifos and spinosad in 2007
Gibraleón (Huelva)	2008	Citrus	Malathion in 2006-2007 and malathion and spinosad in 2008
Castellserà (Lleida)	2009	Apple	Deltamethrin in 2008; methyl-chlorpyrifos and deltamethrin in 2009
Sagunt (València)	2009	Citrus	Spinosad since 2008
	2010	Citrus	Spinosad since 2008
	2015	Citrus	Spinosad since 2008
Albal (València)	2015	Citrus	Spinosad in 2015
Algarrobo Costa (Málaga)	2015	Cherimoya	No treatment in the last years (experimental field)

Table S2. Susceptibility to a discriminating concentration of spinosad of field populations and a control laboratory strain (C) of *Ceratitis capitata*

Population	Year	Mortality % ± SE (n) [(†)]	
		No treatment	Spinosad (1ppm)
Laboratory (C)	-	4 ± 2 (131)	88 ± 3 (153)
La Vila Joiosa (Alicante)	2007	9 ± 9 (44)	85 ± 2 (60)
Almuñécar (Granada)	2007	18 ± 5 (45)	85 ± 9 (60)
Valle de Río Verde (Granada)	2007	0 (30)	87± 6 (60)
Alcanar (Tarragona)	2007	7 ± 3 (100)	78 ± 3 (88)
Muro (Mallorca)	2007	3 ± 3 (40)	77 ± 8 (44)
Gibraleón (Huelva)	2008	11 ± 6 (44)	77 ± 8 (44)

[(†)] Feeding assays (48 h) performed with Spintor Cebo. The total number of flies tested indicated between brackets (3–4 replicates of 10–15 flies each). Mortality in field populations was not significantly different from that of the laboratory C strain (P <0.05, Dunnett's test, using arcsine square root transformation of mortality data from spinosad treatment, corrected for natural mortality in untreated animals using Abbott's formula).

Table S3. Correspondence of the different alleles detected in JW-100s and the mutations included in each allele.

		MUTATIONS		
		3aAG>AT	3aQ68*	K352*
ALLELES	+	✗	✗	✗
	Ccα6$^{3aAG>AT}$	✓	✗	✗
	Ccα6^{3aQ68*}	✗	✓	✗
	Ccα6$^{3aQ68*-K352*}$	✗	✓	✓

Table S4. Variations on allele frequency during different generations of JW-100s. All individuals analysed are survivors under the indicated spinosad concentrations/doses.

Generation	Spinosad treatment [†]	n[‡]	Allele frequency			
			+	*Ccα6*$^{3aAG>AT}$	*Ccα6*^{3aQ68*}	*Ccα6*$^{3aQ68*-K352*}$
F0	nt	5	1	-	-	-
	1-5[§] ppm	37	1	-	-	-
F25	nt	30	0.32	0.3	0.38	-
	6 ppm	20	0.05	0.43	0.52	-
F29	nt	30	-	0.45	0.55	-
	240 ppm	20	-	0.2	0.8	-
F48	nt	24	-	-	0.021	0.979
	100 μg/g	24	-	-	-	1
F85	nt	48	-	-	0.042	0.958
	100 ppm	15	-	-	0.033	0.967

[†] Spinosad was administered by feeding (concentration in ppm) on F0, F25, F29 and F85, and by topical application (dose in μg/g) on F48. No treatment (nt).
[‡] Number of flies genotyped
[§] Includes 26 survivors at 1ppm, 8 survivors at 3 ppm and 3 survivors at 5 ppm
"+" refers to the alleles bearing none of the mutations described (AG>AT, Q68* or K352*)
"-" means that the frequency detected for a specific genotype was 0

Figure S1. Map of Spain indicating the location of the field populations analysed in
lines delimitate the different Autonomous Communities in Spain.

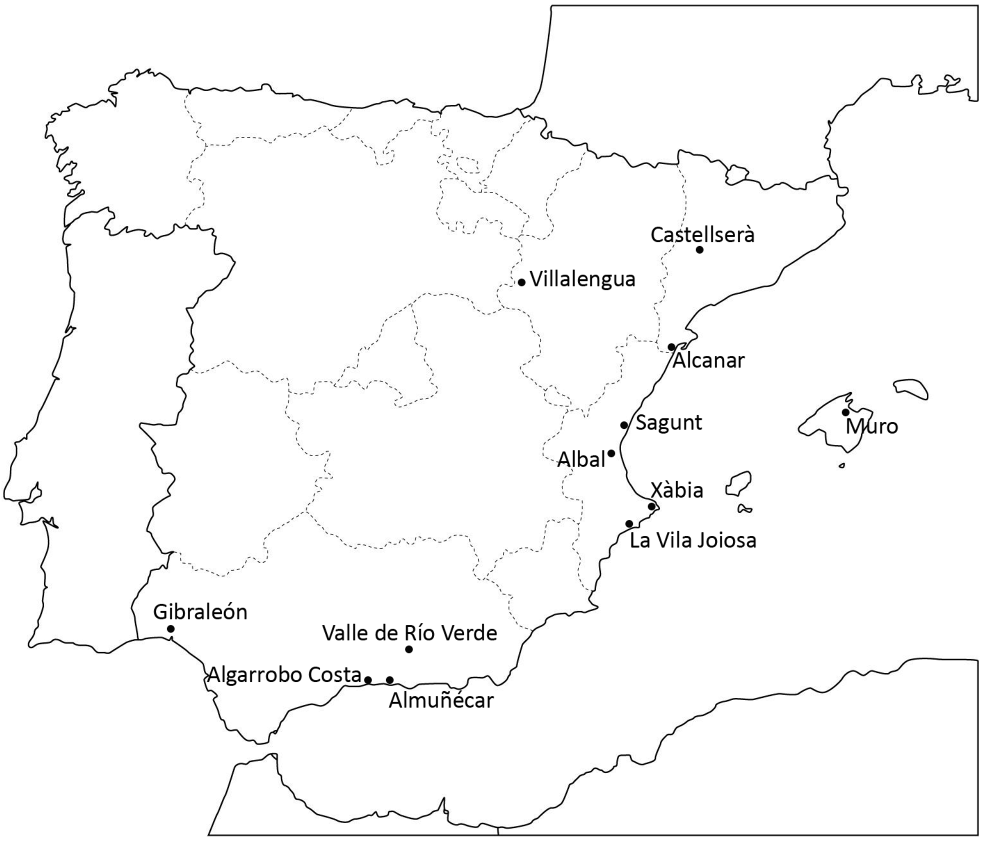

Figure S2. Representative images of band patterning observed after different PCR methods used to detect the three *Ccα6* mutations observed in this study. (A) Gel electrophoresis obtained after the PCR-RFLP method to detect the presence of the K352* mutation. Three different patterns of bands corresponding to a K352*/K352* homozygous, a +/K352* heterozygous and a +/+ homozygous are observed. (B) Gel electrophoresis showing the band patterning observed after multiplex PCR-1, that allows the detection of the two mutations (Q68* and AG>AT) present on exon 3a. (C) Gel electrophoresis of a representative multiplex PCR-2 used to discriminate between heterozygous +/Q68* and homozygous Q68*/Q68* individuals. (D) Gel electrophoresis of a representative multiplex PCR-3 used to discriminate between heterozygous +/AG>AT and homozygous AG>AT/AG>AT individuals.

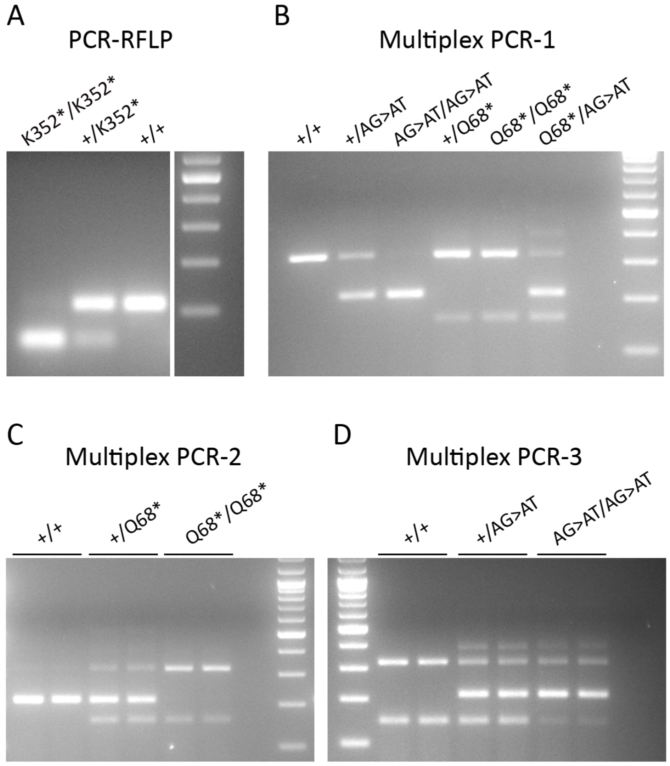

Figure S3. Genomic sequence of the wild-type *Ccα6* exon 3a (blue box) and the flanking intronic regions. Localization and direction (forward or reverse) of the oligonucleotides used for multiplex PCRs are indicated. Bold nucleotides indicate the position of the different exon 3a mutations described in this study.

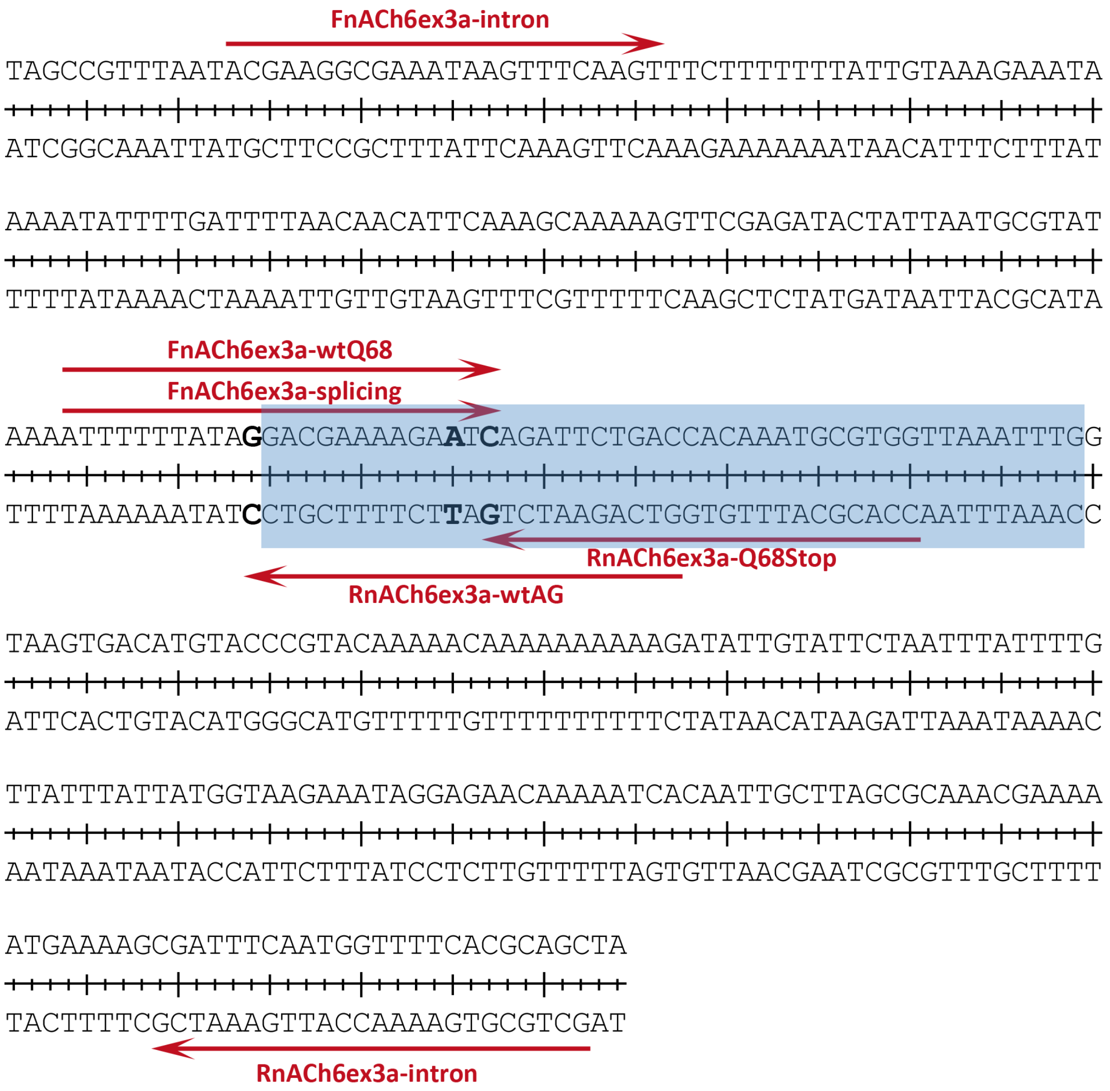

Figure S4. Sequencing chromatograms obtained from individuals bearing *Ccα6* mutations. (A) Alignment of the wild-type sequences of exon 3a, exon 3b and the chromatograms obtained after sequencing the cDNA and the genomic region of exon 3a and exon 3b of a JW-100s individual (F81) homozygous for the Q68* mutation. The double traces on cDNA chromatogram indicate the expression of exon 3a and exon 3b. (B) Alignment of the wild-type sequence of a fragment of exon 10 and the chromatogram obtained after sequencing the cDNA of a JW-100s individual (F81) homozygous for the K352* mutation. (C) Alignment of the *wild-type* sequences of exon 3a and its flanking intronic region, with the chromatogram obtained after sequencing the genomic DNA of a JW-100s individual (F25) homozygous for the AG>AT mutation and the associated A200T mutation. Mutations are highlighted with red boxes.

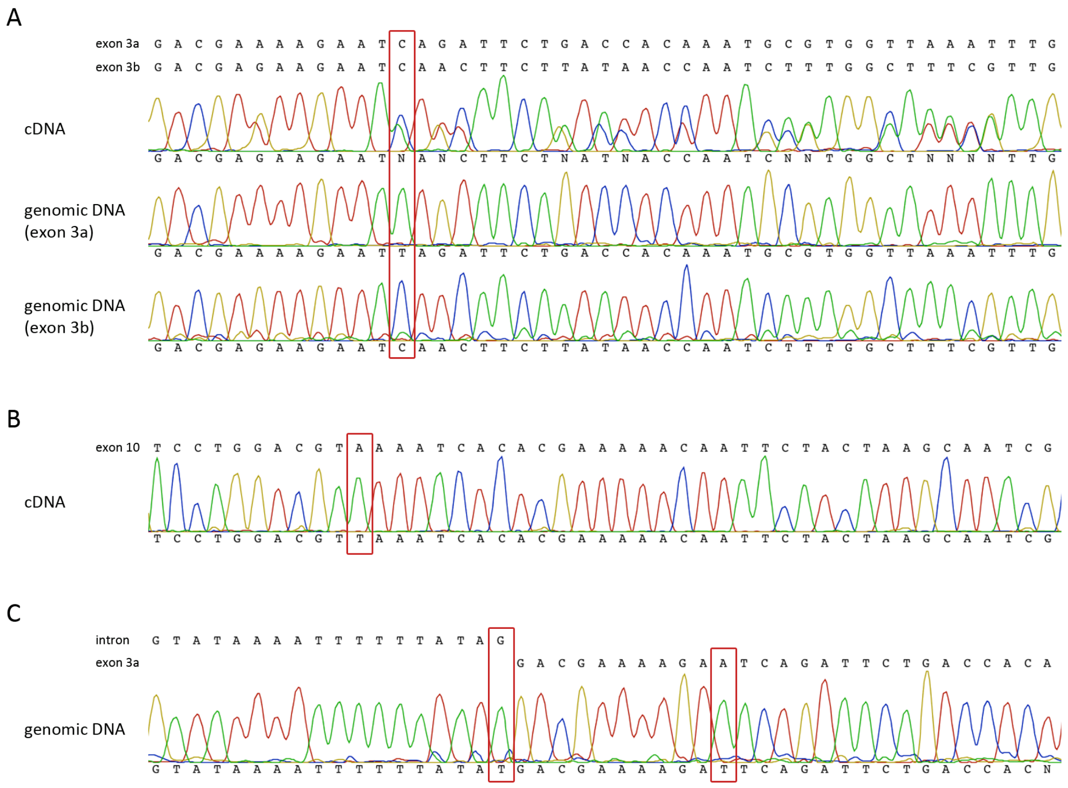

Supplement: Supplementary file 1 — Supplementary Information [file 41598_2019_38681_MOESM1_ESM.pdf]
